# Development of an Intergeneric Conjugal Transfer System for Xinaomycins-Producing *Streptomyces noursei* Xinao-4

**DOI:** 10.3390/ijms150712217

**Published:** 2014-07-09

**Authors:** Feng-Hui Sun, Di Luo, Dan Shu, Juan Zhong, Hong Tan

**Affiliations:** 1 Key Laboratory of Environmental and Applied Microbiology, Chengdu Institute of Biology, Chinese Academy of Sciences, No. 9 Section 4, Renmin Nan Road, Chengdu 610041, China; E-Mails: sunfenghui_83@hotmail.com (F.-H.S.); lddd@163.com (D.L.); whosecats@163.com (D.S.); zhongjuan@cib.ac.cn (J.Z.); 2 Environmental Microbiology Key Laboratory of Sichuan Province, No. 9 Section 4, Renmin Nan Road, Chengdu 610041, China; 3 University of the Chinese Academy of Sciences, No. 19A Yuquan Road, Beijing 100049, China

**Keywords:** *Streptomyces noursei*, xinaomycins, intergeneric conjugation, glycosyltransferase

## Abstract

To introduce DNA into *Streptomyces noursei* xinao-4, which produces xinaomycins, we explored an intergeneric conjugal transfer system. High efficiency of conjugation (8 × 10^−3^ exconjugants per recipient) was obtained when spores of *S. noursei* xinao-4 were heat-shocked at 50 °C for 10 min, mixed with *Escherichia coli* ET12567 (pUZ8002/pSET152) in the ratio of 1:100, plated on 2CMY medium containing 40 mmol/L MgCl_2_, and incubated at 30 °C for 22 h. With this protocol, the plasmids pKC1139 and pSET152 were successfully transferred from *E. coli* ET12567 (pUZ8002) with different frequencies. Among all parameters, the ratio of donor to recipient cell number had the strongest effect on the transformation efficiency. In order to validate the above intergeneric conjugal transfer system, a glycosyltransferase gene was cloned and efficiently knocked out in *S. noursei* xinao-4 using pSG5-based plasmid pKC1139.

## 1. Introduction

*Streptomyces* are soil-dwelling Gram-positive bacteria, and produce various bioactive molecules including half of microbial origin antibiotics employed in the field of medicine and agriculture [1,2]. Peptidyl nucleoside antibiotics are a group of secondary metabolites with similar chemical structures, which possess impressive antitumoral, antiviral, antibacterial, and antifungal activities [3,4]. The biosynthesis of peptidyl nucleoside antibiotics has been actively pursued due to their unique structures and clinically-potential applications recently. In the past few years, the biosynthetic gene clusters had been identified for nikkomycin [5], mildiomycin [3,6], gougerotin [7], blastididin S [8], puromycin [9], polyoxin [10], and streptothricin F [11]. Xinaomycins are a group of *Streptomyces*-derived peptidyl nucleoside antibiotics with potential antibacterial [12], antifungal [13], and antiviral [14,15] activities produced by *S. noursei* xinao-4 that has been isolated by our lab [16]. In order to improve xinaomycins production and get the highly pure xinaomycins, it is desirable to study the xinaomycins biosynthetic gene clusters. However, the fact that the genetic manipulation system of *S. noursei* xinao-4, such as gene cloning, transformation of vectors, and gene deletion has not been developed retards its molecular genetic studies. The present study aimed at the development of an efficient transformation system for the *S. noursei* xinao-4 in order to carry out the genetic manipulation of this strain.

There are three genetic transformation systems available that have been used to transfer foreign plasmids into *Streptomyces*. The protoplast transformation and electroporation techniques have been widely used [17,18]. Unfortunately, these two methods are relatively inefficient mainly because of the strain-specific conditions for protoplast formation and regeneration, and also the presence of different restriction-modification systems in each strain. Intergeneric conjugation between *Escherichia coli* and *Streptomyces* which was firstly reported in 1989 [19], was proved to be a reliable method. Especially the employment of methylation-deficient *E. coli* as donor and the construction of various *E. coli* shuttle vectors [20] improved this method, so that it was used widely in the *Streptomyces* [1,21,22,23,24,25,26]. Although transformation systems for several *Streptomyces* strains have been developed since 1989 [1,21,22,23,24,25,26], there is still no universal protocol applicable to all *Streptomyces* strains. We have carried out an intergeneric conjugation according to the standard procedure described by Kieser *et al.* [22]. Unfortunately, few exconjugants harboring integrative plasmid pSET152 were received at low efficiency (about 10^−6^), and no deletion mutants (conducted by suicide vector pOJ260) was obtained.

In this study, we report a reliable genetic system for *S. noursei* xinao-4 by optimization of transformation conditions including conjugal medium, heat-shock, and ratio of donor to recipient cell number, as well as incubation time of the conjugation plates. The conjugation efficiency of integrative and self-replicative plasmids and the effect of these two plasmids on xinaomycins production were also evaluated. A glycosyltransferase (*glt*) gene was successfully disrupted based on the temperature sensitive plasmid pKC1139 with the established transformation system. We developed an efficient intergeneric conjugation procedure to introduce the plasmid DNA into *S. noursei* xinao-4 that may form the basis of gene functional analysis.

## 2. Results and Discussion

### 2.1. Antibiotic Tolerance of S. noursei Xinao-4

In order to establish an efficient transformation system, we initially studied the antibiotic tolerance of *S. noursei* xinao-4. The results showed that the organism was sensitive to thiostrepton (at concentration >6.25 µg/mL), neomycin (>12.5 µg/mL), kanamycin (>25 µg/mL), and apramycin (>12.5 µg/mL). The *S. noursei* xinao-4 shows some background growth on YMS media including <25 µg/mL spectinomycin. It is sensitive to apramycin and thiostrepton at concentrations >12.5 µg/mL and 6.25 µg/mL respectively. Thus, these two antibiotics are favorable for selection of exconjugants.

*E. coli* ET12567 (pUZ8002) containing pSET152 was used as the donor in the following optimization of intergeneric conjugation. Several parameters that might affect the efficiency of conjugation were evaluated, e.g., mating solid media, heat-shock, ratio of donor to recipient cell number, and incubation time of the conjugation plates.

### 2.2. Optimization of Solid Media for Exconjugants Regeneration

Previous studies showed that different conjugal medium played a significant role for the conjugal efficiency and the appropriate conjugal medium varied between different *Streptomyces* strains [1,24]. To select the appropriate medium for intergeneric conjugation, five agar media (MS, ISP4, 2CMY, YMS and TSA) containing 10 mmol/L MgCl_2_ were assessed as conjugative medium. Among them, MS, ISP4 and 2CMY has been used as conjugal medium for several *Streptomyces* [1,21,25,26,27,28]; TSA is usually applied in the growth of *Streptomyces* species [1,22]; and YMS is the suitable medium for the growth and spore formation of *S. noursei* xinao-4. *S. noursei* xinao-4 grow and sporulate fast within 7 days on YMS agar at 28 °C. The results (data not shown) showed that TSA and YMS were not suitable for conjugation because *E. coli* outgrew over *S. noursei* xinao-4 during overnight incubation, with few exconjugants (about 1 × 10^−6^) on these two media. When compared to the conjugation frequency of MS medium and ISP4 medium, the conjugation frequency of 2CMY medium increased 1.8 and 1.3 times, respectively, reaching (6.04 ± 0.68) × 10^−5^. Thus, 2CMY medium was proved to be the most appropriate medium for intergeneric conjugation.

**Figure 1:**
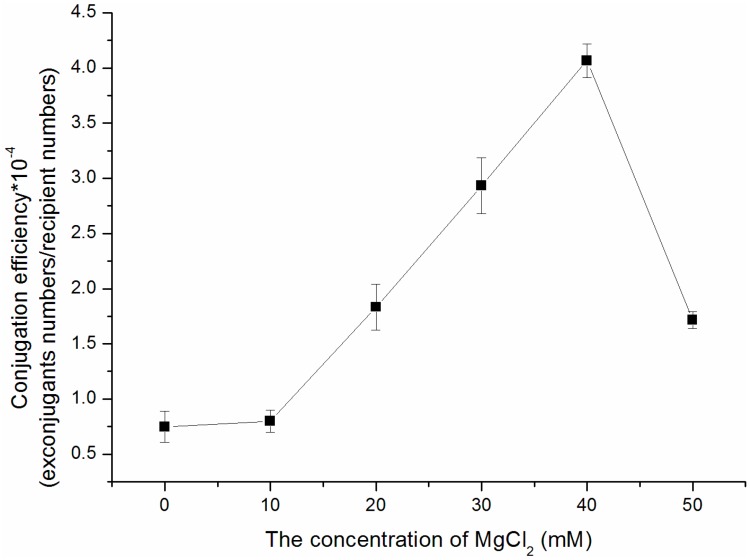
Effect of the concentration of MgCl_2_ on conjugation efficiency with *E.* *coli* ET12567 (pUZ8002/pSET152) on 2CMY agar medium.

Generally, MgCl_2_ has been added to the conjugation medium to improve the efficiency of conjugation [22,23]; however, optimal concentration of MgCl_2_ differed between *Streptomyces* strains. In our study, the conjugation medium 2CMY was further optimized with a supplement of 0, 10, 20, 30, 40, 50 mM of MgCl_2_, respectively. As the results shown (Figure 1), conjugation efficiency was further increased, in the presence of higher concentrations of MgCl_2_. The highest conjugation efficiency was achieved in the presence of 40 mM MgCl_2_. The conjugation efficiency decreased dramatically, when the concentration was higher than 40 mM MgCl_2_, e.g., 50 mM. Our results revealed that 40 mM MgCl_2_ appeared to be the optimal concentration for conjugation of *S. noursei* xinao-4.

### 2.3. Effect of Heat-Shock on the Conjugation Efficiency

Heat-shock of the *Streptomyces* spores has been generally recommended before mating with *E. coli* donors [19,21,22]. To determine the optimal temperature for heat treatment, we evaluated the effect of heat treatment with different temperature on the spore viability and conjugation efficiency. As shown in Table 1, we found that the efficiency of conjugation increased along with temperature rising until reaching 50 °C, but decreased rapidly above 50 °C. The highest efficiency was achieved using a 50 °C heat shock, and was about 10 times that obtained at room temperature. As expected, the spore viability decreased as the temperature of heat-treatment increased, and the spores lost their viability quickly when they were incubated at 55 °C for 10 min. Apparently, the conjugation efficiency was not consistent with spore viability, indicating that the effect of heat-treatment on conjugation efficiency might be attributed to the temporal reduction of the restriction enzyme activities, other than increasing the viability of spores [29,30].

**Table 1:** Effect of heat treatment of *S. noursei* xinao-4 spores on spore viability and conjugation efficiency with *E.* *coli* ET12567 (pUZ8002/pSET152) on 2CMY agar medium containing 10 mM MgCl_2_.

Temperature (°C)	Viability (%)	Conjugation Frequency
Room temperature	100	(4.87 ± 0.31) × 10^−5^
40	95.5	(8.0 ± 0.56) × 10^−5^
45	69.87	(1.96 ± 0.29) × 10^−4^
50	56.12	(3.47 ± 0.21) × 10^−4^
55	17.97	(7.1 ± 0.45) × 10^−5^

**Figure 2:**
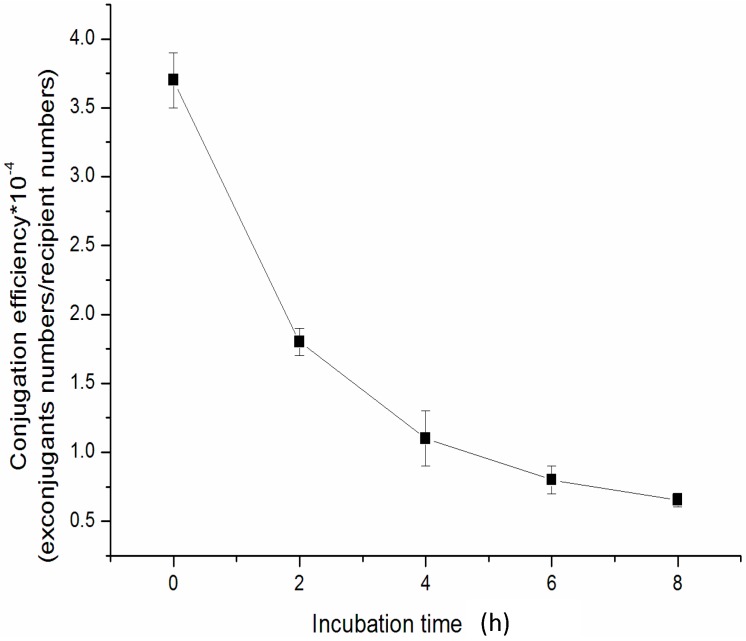
Effect of incubation of heat-treated spores on conjugation efficiency with *E. coli* ET12567 (pUZ8002/pSET152) on 2CMY agar medium containing 10 mM MgCl_2_.

Incubation of heat-shocked spores prior to mixing with *E. coli* was recommended for many *Streptomyces* strains [23,31]. Therefore, the effect of incubation time of heat-shocked spores on the conjugation efficiency was also investigated. As shown in Figure 2, the longer incubation of heat-shocked spores was, the fewer exconjugants were received. Thus, we treated the *S. noursei* xinao-4 spores at 50 °C for 10 min without further cultivation for conjugation.

### 2.4. Effect of Donor-to-Recipient Ratio on Conjugation Efficiency

The ratio of donor cell number to recipient spore number was a crucial parameter in the intergeneric conjugation of *Streptomyces*. One mL of *S. noursei* spores at a concentration of 10^7^ per mL, heat-treated at 50 °C for 10 min and a range of *E. coli* ET12567 exponential cells (10^7^–10^10^) were mixed, and the conjugation efficiency was evaluated. As shown in Table 2, few exconjugants were obtained when the number of donor cells was less than 10^7^. When the number of the donor cells was up to 10^9^, the conjugation efficiency reached the highest efficiency of (2.3 ± 0.24) × 10^−3^, which is 34 times greater than that obtained with 10^7^ donor cells. In contrast with the results of *Streptomyces coelicolor* [21] and *Streptomyces nodosus* [25], where the donor-to-recipient ratio did not affect the conjugal efficiency, our results indicated that the maximum efficiency was received with a certain donor-to-recipient ratio of 100:1. Similar results had been observed in *Kitasatospora setae* [32], *Streptomyces lincolnensis* [24], and *Streptomyces diastatochromogenes* 1628 [31], *Streptomyces natalensis* [33] in which a certain ratio of donor to recipient cell number was required to achieve highest frequency.

**Table 2:** Effect of donor-to-recipient on conjugation efficiency with *E. coli* ET12567 (pUZ8002/pSET152) on 2CMY agar medium containing 10 mM MgCl_2_.

Donor Numbers (×10^7^)	Conjugation Frequency
1	(6.59 ± 0.29) × 10^−5^
10	(5.5 ± 0.27) × 10^−4^
100	(2.3± 0.24) × 10^−3^
1000	(4.4 ± 0.2) × 10^−4^

### 2.5. The Effect of Incubation Time of Mix-Culture on the Conjugation Efficiency

**Figure 3:**
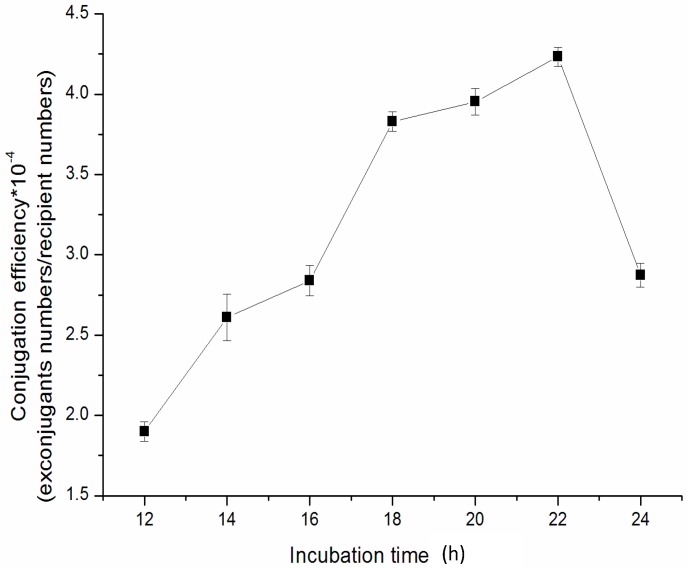
Effect of incubation time of mixed culture on conjugation efficiency with *E. coli* ET12567 (pUZ8002/pSET152) on 2CMY agar medium containing 10 mM MgCl_2_.

The intergeneric conjugation, especially, the plasmid transfer, takes place during the incubation of the mixed culture of *E. coli* and *Streptomyces*, stressing the importance of incubation time of mix-culture of these two strains. If the incubation time is too short, the conjugation cannot be accomplished, resulting in lower conjugation efficiency. On the contrary, the longer the incubation time, the greater is the probability of false positives. As shown in Figure 3, more exconjugants were received with an incubation time prolonged from 12 to 22 h. There was little difference in conjugation efficiency among 18, 20 and 22 h incubation time. Nevertheless, when the incubation time was extended to 24 h, there were many false positive exconjugants on the plate, which hampered the selection of the positive exconjugants. Our results indicated that the best incubation time of mix-culture plates was 18–22 h.

### 2.6. Conjugal Transfers of Integrated and Self-Replicative Plasmid into S. noursei Xinao-4

In order to assess the availability of the present intergeneric conjugal transfer system, two common *Streptomyces* plasmids, pKC1139 and pSET152 were transferred into *S. noursei* xinao-4. Both plasmids used in these transformation experiments contain the origin of transfer, *oriT* from plasmid RP4 allowing mobilization of the single strand during conjugation from *E. coli* ET12567 into *Streptomyces*. The plasmids were successfully transferred into *S. noursei* xinao-4 albeit with different frequencies. The frequency of plasmid pKC1139 was lower than that of integrative plasmid pSET152. The conjugation frequency was up to (8.0 ± 0.14) × 10^−3^ per recipient for *S. noursei* xinao-4 under optimal conditions with integrative plasmid pSET152, while the frequency of plasmid pKC1139 was (3.5 ± 0.24) × 10^−5^.

The PCR experiments confirmed that all randomly chosen exconjugants displayed a 519 bp band of the apramycin resistance gene, demonstrating the presence of the plasmid (data not shown). The exconjugants were stable after five successive sub-cultivations, indicating that this intergeneric conjugation strategy can be widely applied for genetic research in the future. All exconjugants did not differ from the wild-type strain, *S. noursei* xinao-4, in their ability to produce xinaomycins. Besides that, we did not observe any changes in the sporulation or morphology of the exconjugants on YMS plates when compared to the wild-type strain (data not shown).

### 2.7. Validation of Conjugation System: A Case Study

Suicide vectors and pSG5-based temperature-sensitive plasmids were widely used for gene knock-out in *Streptomyces*. In order to compare these two kinds of plasmids for deletion in *S. noursei* xinao-4 and validate the established intergeneric transformation system, a 3230 bp fragment of putative glycosyltransferase gene (*glt*) was cloned from its genomic DNA. It was reported that glycosyltransferase can transfer glycosyl into various acceptors, such as oligosaccharides, lipids, proteins, nucleic acids and innumerous natural products [34,35], such as in the biosynthesis of nucleoside antibiotics, cytosinpeptidemycin *Streptomyces ahygroscopicus* [36] and amicetin in *Streptomyces vinaceusdrappus* [35,37]. This enzyme may be involved in xinaomycins biosynthesis. We cloned a glycosyltransferase gene (*glt*) from the genomic DNA of *S. noursei* xinao-4 by degenerate primers. The obtained fragment shows high sequence similarity with many glycosyltransferase genes from other *Streptomyces*. The two vectors pOJ2601 and pKC1139-1 for *glt* knock-out were constructed as above.

About one single crossover exconjugant was obtained per 10^10^ spores using suicide vector pOJ2601. Unfortunately, we failed to receive any double-crossover mutants by pOJ2601 directly. One of the single-crossover exconjugants was allowed three rounds of sporulation on YMS agar without antibiotic selection and its progeny were screened for double-crossover mutants. About one thousand colonies were screened, three of which were confirmed to be double-crossover mutants. The low efficiency of double-crossover mutants with suicide vector may be attributed to the short homologous arm (about 1 kb) present in plasmid pOJ2601.

**Figure 4:**
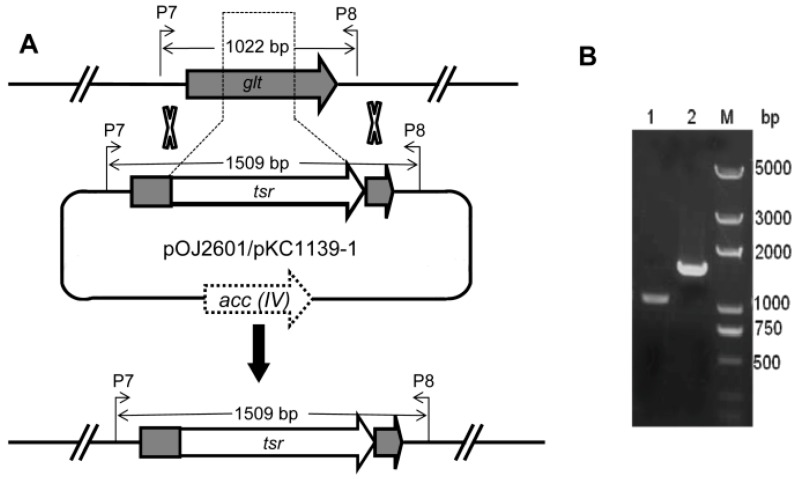
Inactivation of glycosyltranferase gene by gene deletion. (**A**) Construction of deletion mutants of *glt*; and (**B**) Verification of the double crossover mutants by PCR from wild-type (1022 bp) and mutant strain (1509 bp). Lane 1: wild-type strain; lane 2: mutant strain; M: marker.

About 10^3^ transformants were obtained using the plasmid pKC1139-1, twelve exconjugants of which were verified by PCR and further incubated for sporulation at 30 °C. The harvested spores were spread on YMS plate containing thiostrepton and incubated at 37 °C for 7 days to eliminate the free plasmids from the host cells. The spores were collected and spread on YMS agar containing thiostrepton or apramycin to screen the double-crossover mutants. Five hundred colonies were chosen, and nineteen double-crossover mutants of which were obtained. Three mutants were randomly selected and verified by PCR. As shown in Figure 4, mutants have 571 bp deletions of *glt* gene and showed a 1509 bp fragment with the gltp7 and gltp8 as the primer.

When compared to wild-type strain, *S. noursei* xinao-4, the *glt* mutants exhibited no visible change on xinaomycins synthesis and morphogenesis by shake flask fermentation in BL medium, suggesting that deletion of *glt* had no effect on xinaomycins production under the condition we tested. Nevertheless, the *glt* gene was disrupted successfully by above protocol, and 22 deletion mutants were obtained with these two plasmids. Apparently, temperature-sensitive plasmids had a greater chance to acquire deletion mutants, which provided a practical tool for further genetic manipulation of *S. noursei* xinao-4.

## 3. Experimental Section

### 3.1. Strains and Plasmids

*E. coli* DH5α was used as general host for cloning and plasmid reproduction [38]. Methylation-deficient *E. coli* ET12567 (*dam-13::Tn 9*, *dcm-6*, *hsdM*, *hsdS*) [39], harboring plasmid pUZ8002 [40] was used as the donor for conjugation experiments. *S. noursei* xinao-4, the xinaomycins producer, was maintained in our lab and used as recipient in intergeneric conjugation. The replicative vector pKC1139 [20] contains pSG5 origins of replication and intergrative vector pSET152 [20] contains *int-attp* fragments of actinophages φC31, were used as conjugative vectors to transfer from *E. coli* ET12567 to *S. noursei* xinao-4. pEASY-Blunt Zero (TransGen Biotech, Beijing, China) vector was used for cloning of the PCR products. The conjugative plasmids pOJ260 [20] and pKC1139 were used to construct the deletion vectors. The plasmid pWHM3 [41] served as the template to amplify the thiostrepton resistance gene. The *glt* deletion vectors pOJ2601 and pKC1139-1 were constructed as described below. *Bacillus cereus* was used as indicator for bioassay of xinaomycins.

### 3.2. Media and Culture Condition

*E. coli* strains were grown in Luria-Bertani (LB) medium supplemented with appropriate antibiotics at 200 rpm in a shaking incubator at 37 or 30 °C as required [42]. *S. noursei* xinao-4 was routinely grown on YMS agar at 28 °C. The spores of *S. noursei* xinao-4 were achieved on YMS medium at 30 °C for 7 days, and then scraped and used as recipients. Mycelia for DNA extraction were grown in TSB broth containing 1% glycine (*w*/*v*) at 28 °C for 2 days [22]. MS [43], ISP4 [44], 2CMY [45], Tryptone soya agar (TSA) [22] and YMS [46] plates were used as the conjugative media. Apramycin (50 µg/mL), ampicillin (50 µg/mL), chloramphenicol (25 µg/mL), kanamycin (50 µg/mL), thiostrepton (25 µg/mL) and nalidixic acid (50 µg/mL) was added as required.

### 3.3. Construction of Plasmids for Gene Deletion

The constructions of vectors for gene inactivation were carried out according to Shevchuk *et al.* [47]. A 1058 bp fragment designated as tsr, including the coding region of thiostrepton resistance gene and its promoter sequence was amplified from the plasmid pWHM3 with tsrF (5'-TGATCAAGGCGAATACTTCAT-3') and tsrR (5'-TCATCACTGACGAATCGAGGT-3'). A 1216 bp fragment designated glt1, encompassing the region upstream of *glt* gene and some of its encoding region, was amplified from the *S. noursei* xinao-4 genomic DNA with primers gltp1 (5'-CTCGCCCATGTTCCCGTAC-3') and gltp2 (5'-ATGAAGTATTCGCCTTGATCAACCGCCGTTCTTCTCGTGCC-3'). A 1236 bp fragment designated glt2, encompassing some of *glt* gene encoding region and the region downstream of this gene, was amplified with primers gltp3 (5'-ACCTCGATTCGTCAGTGATGATCTTCCTGGCCTCCACCCTCTA-3') and gltp4 (5'-GGCATCCGGTTGCTGTTC-3') (the sequence overlapping the tsr fragment is underlined). The obtained three fragments were ligated together according to the long multiple fusion (LMF) method [47]. The PCR reactions (50 µL) contained 0.5 U KOD-Plus-Neo DNA Polymerase (Toyobo, Shanghai, China), 5 µL 10× PCR Buffer for KOD-Plus-Neo, 1 mM of MgCl_2_, 200 ng of template DNA, 200 µM of each dNTP, 0.2 µM of each primer, and 2.5 µL of DMSO. PCR was performed as follows: after initial denaturation at 94 °C for 5 min, 30 cycles PCR reaction were performed at 94 °C for 30 s, 62 °C for 30 s and 68 °C for 1 min.

A 3409 bp fragment including the 1141 bp upstream homologous arm, the 1058 bp thiostrepton resistance cassette, and the 1210 bp downstream homologous arm, was obtained with the primers gltp5 (5'-CGCGGATCCGGGCAGTTCCTCGATGTGGGTG-3') (with a *Bam*HI site, underlined) and gltp6 (5'-CCCAAGCTTGCTGTTCTGGTACGGCGTCAACC-3') (with a *Hin*dIII site, underlined). The obtained 3409 bp fragment was cloned to pEASY-Blunt Zero (TransGen Biotech, Beijing, China), and then digested with *Bam*HI and *Hin*dIII. The resulting fragment was ligated to pOJ260 or pKC1139, which had been cut with *Bam*HI and *Hin*dIII endonuclease, yielding the *glt* deletion vector pOJ2601 and pKC1139-1.

### 3.4. Intergeneric Conjugation Procedure and Analysis of Exconjugants

Intergeneric conjugation between *E. coli* and *S. noursei* xinao-4 was carried out as described by Kiser *et al.* [22] with some modification. A culture of the donor *E. coli* ET12567 (pUZ8002) containing conjugative plasmid was grown to an OD600 of 0.4–0.6 with the appropriate antibiotics. To remove the antibiotics, the cells were collected and washed twice with LB, and then suspended in 0.1 volume of LB medium. *S.* *noursei* xinao-4 spores were collected with 2× YT broth after growth for seven days on YMS medium at 30 °C. Aliquots of fresh *S. noursei* xinao-4 spores were collected, washed twice and suspended in 2× YT broth at a concentration of 10^7^ per mL. The *S.* *noursei* xinao-4 spores were heated at 40–50 °C for 10 min, incubated at 37 °C for 0–8 h and served as recipients. Donors and recipients were mixed and spread on MS, ISP4, 2CMY, TSB and YMS plates and grown for 12–24 h at 30 °C. The effect of MgCl_2_ concentration (0–50 mM) on conjugal efficiency was also evaluated. After incubation, the plates were covered with 1 mL water containing 0.625 mg nalidixic acid and 0.625 mg apramycin or thiostrepton as required, and incubated at 30 °C for 3–7 days, until the exconjugants appeared.

### 3.5. Analysis of Exconjugants

Total DNA of exconjugants was prepared according to the method of Kieser *et al.* [22]. A 519 bp fragment was amplified by PCR with the specific primers AAC-F (5'-TGGGCCACTTGGACTGAT-3') and AAC-R (5'-CCGACTGGACCTTCCTTCTG-3') within the apramycin resistance gene, demonstrating the presence of vector pSET152 or pKC1139. The knock-out mutants were verified by PCR with the specific primers gltp7 (5'-CGGAATGGGTACGTGATGG-3') and gltp8 (5'-GCCGAGATGGTGGGAAATG-3') within the homologous arms. The double crossover mutants revealed a 1509 bp fragment, differing from the 1022 bp fragment found in the wild type strain. The PCR reactions (50 µL) contained 2.5 U Easy Taq DNA Polymerase (TransGen Biotech, Beijing, China), 5 µL 10× Easy Taq buffer, 200 ng of template DNA, 250 µM of each dNTP, 0.2 µM of each primer, and 2.5 µL of DMSO. PCR was performed as follows: after initial denaturation at 94 °C for 5 min, 30 cycles PCR reaction were performed at 94 °C for 30 s, 62 °C for 30 s and 72 °C for 1 min.

### 3.6. Fermentations and Bioassay of Xinaomycins

Fermentation was carried out in 250 mL Erlenmeyer flasks containing BL medium (containing glucose 6.0 g/L, corn powder 6.0 g/L, yeast powder 0.5 g/L, soybean 9.0 g/L, KNO_3_ 1.0 g/L, NaCl 3.0 g/L, (NH_4_)_2_SO_4_ 2.0 g/L, CaCO_3_ 2.0 g/L, and MgSO_4_·7H_2_O 0.5 g/L, pH 7.2). The seed culture was cultivated in 40 mL BL medium under 150 rpm, at 28 °C for 36 h. The fermentation culture (100 mL BL medium) was inoculated with 5 mL seed culture, and then incubated at 28 °C, under 150 rpm for 2 days. The fermentation broth was collected and centrifuged at 6000 rpm for 10 min. The liquid supernatant was collected and used to detect the xinaomycins [16].

The bioassay of xinaomycins was carried out with SC medium (peptone 1.5 g/L, beef extract 1.5 g/L, Na_2_HPO_4_ 0.05 g/L, agar 18 g/L) according to the cup-plate method, containing *Bacillus cereus*, a strain that is sensitive to xinaomycins. 30 µL of fermentation broth were added to the oxford cup, and were then incubated for 17 h at 30 °C, and screened visually for growth-inhibition zones [12].

## 4. Conclusions

In conclusion, an efficient intergeneric conjugation system of transferring plasmids from *E. coli* to *S. noursei* xinao-4 was developed. Compared to the previous studies, in which the transformation efficiency is below 10^−3^ [1,26,31,33,48], our study showed high transformation efficiency. The availability of this protocol was confirmed by the 22 double-crossover mutants of glycosyltranferase gene. This procedure lays a good foundation for DNA transformation and mutational analysis of this industrially important strain. Also, it becomes a useful tool for introducing deletions and alterations of the genes responsible for xinaomycins biosynthesis, exploring its natural biosynthetic pathway, understanding the regulation of xinaomycins in more details, and subsequently producing new hybrid derivates.
